# An Application of an Initial Full Value of Vaccine Assessment Methodology to Measles-Rubella MAPs for Use in Low- and Middle-Income Countries

**DOI:** 10.3390/vaccines12091075

**Published:** 2024-09-19

**Authors:** Melissa Ko, Collrane Frivold, Mercy Mvundura, Adam Soble, Christopher Gregory, Hans Christiansen, Mateusz Hasso-Agopsowicz, Han Fu, Mark Jit, Shan Hsu, Jessica Joyce Mistilis, Tiziana Scarna, Kristen Earle, Marion Menozzi-Arnaud, Birgitte Giersing, Courtney Jarrahian, Ahmadu Yakubu, Stefano Malvolti, Jean-Pierre Amorij

**Affiliations:** 1 MMGH Consulting GmbH, 1211 Geneva, Switzerland; 2 PATH, Seattle, WA 98121, USA; 3 UNICEF, New York, NY 10038, USA; 4 UNICEF, 2100 Copenhagen, Denmark; 5 World Health Organization, 1211 Geneva, Switzerland; 6 London School of Hygiene & Tropical Medicine, London WC1E 7HT, UK; 7 School of Public Health, The University of Hong Kong, Hong Kong SAR 999077, China; 8 Gavi, The Vaccine Alliance, 1211 Geneva, Switzerland; 9 The Bill and Melinda Gates Foundation, Seattle, WA 98121, USA; 10 MMGH Consulting GmbH, 8049 Zurich, Switzerland

**Keywords:** vaccines, demand estimation, public health impact assessment, cost-effectiveness, discounted cash flow, costing, modeling, value proposition

## Abstract

Measles and rubella micro-array patches (MR-MAPs) are a promising innovation to address limitations of the current needle and syringe (N&S) presentation due to their single-dose presentation, ease of use, and improved thermostability. To direct and accelerate further research and interventions, an initial full value vaccine assessment (iFVVA) was initiated prior to MR-MAPs entering phase I trials to quantify their value and identify key data gaps and challenges. The iFVVA utilized a mixed-methods approach with rapid assessment of literature, stakeholder interviews and surveys, and quantitative data analyses to (i) assess global need for improved MR vaccines and how MR-MAPs could address MR problem statements; (ii) estimate costs and benefits of MR-MAPs; (iii) identify the best pathway from development to delivery; and (iv) identify outstanding areas of need where stakeholder intervention can be helpful. These analyses found that if MR-MAPs are broadly deployed, they can potentially reach an additional 80 million children compared to the N&S presentation between 2030–2040. MR-MAPs can avert up to 37 million measles cases, 400,000 measles deaths, and 26 million disability-adjusted life years (DALYs). MR-MAPs with the most optimal product characteristics of low price, controlled temperature chain (CTC) properties, and small cold chain volumes were shown to be cost saving for routine immunization (RI) in low- and middle-income countries (LMICs) compared to N&S. Uncertainties about price and future vaccine coverage impact the potential cost-effectiveness of introducing MR-MAPs in LMICs, indicating that it could be cost-effective in 16–81% of LMICs. Furthermore, this iFVVA highlighted the importance of upfront donor investment in manufacturing set-up and clinical studies and the critical influence of an appropriate price to ensure country and manufacturer financial sustainability. To ensure that MR-MAPs achieve the greatest public health benefit, MAP developers, vaccine manufacturers, donors, financiers, and policy- and decision-makers will need close collaboration and open communications.

## 1. Introduction

Despite the availability of a highly effective vaccine and World Health Organization (WHO) regional targets towards eliminating measles and rubella, it is estimated that measles caused over 9 million cases and 136,000 deaths in 2022, while 100,000 infants are born with congenital rubella syndrome each year [1,2]. Historically, low- and middle-income countries (LMICs) have struggled to obtain and maintain high coverage, with global vaccination coverage stagnating around 83% and 74% for measles-containing vaccine first and second doses (MCV1, MCV2), respectively [3]. The precarious situation was highlighted by the COVID-19 pandemic, where a drop of five percentage points in MCV1 (from 2019 to 2021) resulted in 37 large and disruptive outbreaks occurring in 2022, a 68% increase compared to 2021 [3,4].

Public health experts have long suspected that the difficulty in achieving and sustaining high coverage is in part due to the characteristics of current MR vaccines that make it challenging to reach under- and un-vaccinated children [5,6,7,8,9]. The current vaccines were introduced in the 1960s, and since then, minimal changes have occurred to their design. The current vaccine is lyophilized and requires reconstitution to be performed by a trained health worker; the multi-dose vial (MDV) presentation most used in LMICs has high levels of wastage and is heat sensitive.

The use of a microarray patch (MAP) presentation, which consists of microscopic projections that deliver a dry vaccine or drug when applied to the skin, is a potential solution to address the limitations of the current needle and syringe (N&S) presentation.

As a single-dose, ready-to-use presentation, MR-MAPs could offer several potential benefits, such as being easier to use, reducing administration errors, eliminating the requirement of administration by health workers, reducing open vial wastage, and having improved thermostability, which could lead to increasing vaccination reach to populations where under- and un-immunized children reside [10,11,12,13]. All those features could revolutionize how vaccines are delivered; however, the anticipated benefits are still theoretical, owing to their early stage of development.

Additionally, there are several practical challenges related to the successful development of the MR-MAP innovation. For instance, MR-MAPs will be complex to develop, license, and commercialize; they will likely cost more than current MR multi-dose N&S presentations; and before the initial Full Value of Vaccine Assessment (iFVVA), there was a lack of any estimates of their potential health impact and cost-effectiveness [14,15,16].

**Figure 1:**
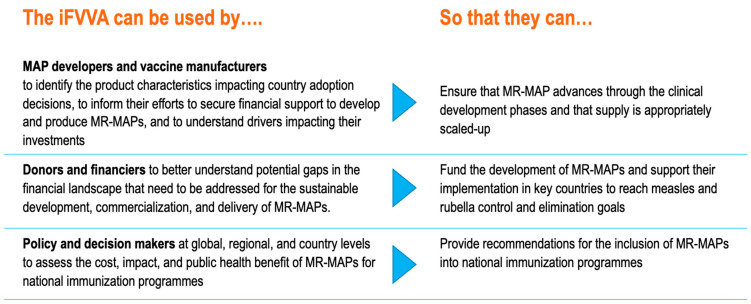
iFVVA uses by target audience.

Even though MR-MAPs only entered phase I trials in 2021 and completed phase I/II trials in 2023, a significant amount of activities were already completed related to the identification of the target product profile (TPP), the identification of the use cases, demand forecasting, and the definition of the MR-MAP business case [15,17,18,19,20]. See Figure S1 for current MR-MAP development timelines. Given the potential high public health impact of MR-MAPs and despite the expectation that the earliest availability of MR-MAPs will be in 2030, we began to develop an initial Full Value of Vaccine Assessment (iFVVA) to integrate all elements in a comprehensive view. The iFVVA utilized current analyses, data, and assumptions and added the additional analyses required to present the different perspectives of the various stakeholders—MAP developers and vaccine manufacturers, donors and financiers, and policy- and decision-makers at global, regional, and national levels—with the goal of defining critical success factors, risks, bottlenecks, and the relevant actions to address those. Figure 1 provides more details on the targeted audience.

The iFVVA also aimed to clearly identify evidence gaps while highlighting potential areas of uncertainty that would require either updated or additional analyses. As MR-MAPs advance in their clinical development and uncertainties begin to be addressed, the iFVVA should be converted into an FVVA that considers the full economic benefit, public health impact, and costs.

## 2. Materials and Methods

The MR-MAP iFVVA was conducted between 2021–2022, adopting a mixed-methods approach that included a rapid assessment of literature, stakeholder interviews and surveys, and various quantitative analyses. An expert advisory group was convened to provide strategic feedback and direction on the iFVVA, including its methodology, results, and key messages (see Table S1 for expert advisory group members). Given that the FVVA methodology provides flexibility, the iFVVA was structured along four sections: (i) articulating the global need for improved MR vaccines and the ability of MR-MAPs to address the MR implementation problems; (ii) estimating the costs and benefits; (iii) describing the pathway from development to delivery; and (iv) identifying the key stakeholders’ actions [21].

An overview of the methodology employed in the first three sections is provided below.

### 2.1. The Global Need for Improved MR Vaccines and Ability of MR-MAPs to Overcome the MR Implementation Problems

This iFVVA section outlined the MR burden and key problem statements for the MR program. While the implementation problems for the MR program are well known, this analysis sought to quantify the level of available evidence and the magnitude of each problem statement. For this, a rapid assessment of literature was conducted, considering key documents from the Vaccine Innovation Prioritization Strategy (VIPS), documents identified by VIPS partners and MR stakeholders, and those retrieved as result of a PubMed search (Table S2 for search terms) [14,22,23,24,25,26,27].

All documents were reviewed, and the problems of programmatic relevance were identified and translated into problem statements, which were documented and organized by theme. We then grouped the problem statements into primary and secondary categories and documented the number of articles citing the problem statements. Next, the product characteristics per the MR-MAP TPP were overlaid to assess if and how MR-MAP characteristics could address the problem statements [28]. Finally, the problem statements were classified as “high/better”, “medium”, or “low/worse” considering the level of evidence available, whether that evidence indicated the likelihood or magnitude of the problem statement occurring.

### 2.2. Estimating the Costs and Benefits of MR-MAP

This section estimated a potential MR-MAP price, its commodity and delivery cost, its public health impact, and its cost-effectiveness. Several existing analyses, such as the MR-MAP use cases and demand forecast, contributed to these goals.

#### 2.2.1. MR-MAP Use Cases and Demand Forecast

The definition of the MR-MAP use cases and the demand forecast were completed prior to the development of the iFVVA and were leveraged [17,18]. These analyses assessed the full spectrum and potential for use of this innovative presentation and hence were developed without any supply, financial, or programmatic constraints.

**Figure 2:**
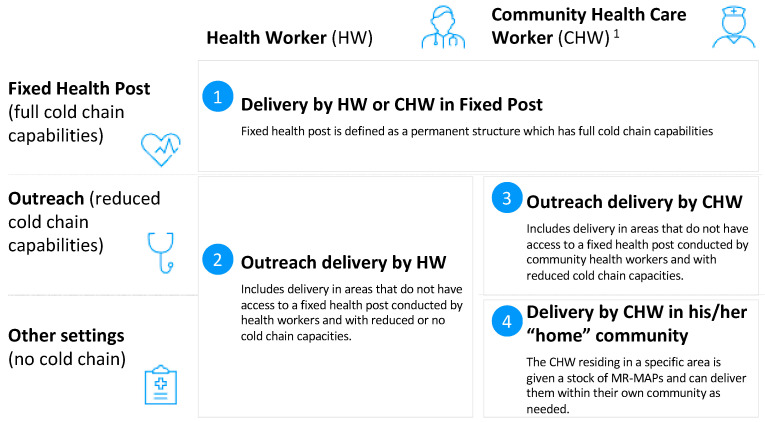
MR-MAP use cases. ^1^ CHW: Community health worker provide health education, referral and follow-up, case management and basic preventive health care and home visiting services to specific communities. They provide support and assistance to individuals and families in navigating the health and social services system. Occupations included in this category normally require formal or informal training and supervision recognized by the health and social services authorities.

The starting point of the analysis was the MR-MAP use cases 1 to 4, as represented in Figure 2 [17,18].

The iFVVA utilized the previously developed MR-MAP demand forecast, which assumes that all countries will introduce and use MR-MAPs on a large scale across use cases 1 through 4 [17]. Without changes in the demand forecast methodology, additional scenarios were developed to better inform the MR-MAP cost and impact analyses that considered different assumptions for MR-MAP country adoption and forecasted coverage growth across the different use cases [17]. Table 1 provides an overview of the forecasting scenarios utilized for the iFVVA. Scenarios 1 and 2 serve as the counterfactual if MR-MAPs never become available.

#### 2.2.2. Benchmarking a MR-MAP Price

To estimate the potential price of MR-MAPs, a price benchmarking approach was used because (i) it is not constrained by confidential price information and can be publicly communicated, and (ii) it used marketed measles and rubella vaccine price data to reflect factors that could influence the manufacturers’ pricing strategies.

The logical cornerstone of the analysis was the assumption that the MR-MAP price would be comparable to the price of MR in a pre-filled syringe (PFS) presentation, as both are single dose presentations with a convenient design that can command the highest mark-up on the cost of goods sold (COGS).

The analysis followed a three-step process: (i) identified appropriate benchmarks where MR vaccines were commercialized in different presentations (MDV, SDV, PFS) across the same procurement archetypes (UN procurement vs. self-procurement and income level) and calculated the price differentials among those; (ii) calculated price ranges for MR across the three different presentations; and (iii) calculated an estimated price range for MR-MAPs considering the procurement archetypes. Benchmark vaccines were selected based on the available price point in the eJRF dataset, where prices existed for different presentations (e.g., MDV, single-dose vial (SDV), or PFS) from the same manufacturer across different markets (e.g., Gavi-supported countries, self-procuring LMICs, upper-middle-income countries (UMICs), and high-income countries (HICs)) and in volumes in excess of 10,000 doses [29,30,31]. See Table S3 for the formula to estimate MR PFS price in Gavi-supported countries.

#### 2.2.3. Estimating the Commodity and Delivery Costs

We estimated the cost per dose administered using the Vaccine Technology Impact Assessment (VTIA) approach, which is an Excel-based model that provides a comparative economic cost assessment of commodity and delivery costs for a current vaccine presentation compared with an innovative presentation [32,33]. The cost assessment was conducted from the health system perspective for a birth cohort vaccinated in 73 LMICs comparing the current 1-, 5- or 10-dose MR N&S as in use by each country to MR-MAPs. All costs are reported in 2021 US$ (US dollars).

Commodity costs included the costs of the (i) vaccine and (ii) immunization supplies. For the vaccine cost, we incorporated the wastage-adjusted vaccine costs, assuming wastage rates of 40% for RI and 10% for supplementary immunization activities (SIAs) [34]. Immunization supplies include auto-disabled (AD) administration syringes, reconstitution syringes, and safety boxes. International freight costs were calculated assuming 3% of the vaccine price.

Delivery costs included (i) vaccine storage or cold chain cost, (ii) transport costs, (iii) human resource (HR) costs of logistics personnel, health workers/vaccinators, and (iv) outreach costs related to additional travel time, transport costs, and per diems to locations outside of the health facility. For SIAs, we included SIA operational costs, assumed to be $1 per dose [35].

To explore the uncertainties in the MR-MAP product attributes, MR-MAP profiles were developed considering variations in estimated price per dose, cold chain volume, and HR time for administration, resulting in four different profiles. We estimated the commodity and delivery costs separately for each MR-MAP profile considering potential use in a controlled temperature chain (CTC) (ability to tolerate ambient temperatures of +40 °C for a minimum of 3 days) [36]. See Table 2 for key assumptions and Table S4 for the additional assumptions used to estimate commodity and delivery costs.

#### 2.2.4. Estimating the Potential Health Impact and Cost Effectiveness

Using the use cases and the demand forecasting scenarios, we estimated the potential public health impact and cost effectiveness of MR-MAPs. The detailed methodology and findings were published [37]. In brief, for public health impact, we used an age-structured dynamic model of measles transmission and vaccination to project measles cases, deaths, and disability-adjusted life years during 2030–2040 [37]. For cost effectiveness, the costs were calculated based on the ingredients approach, including direct costs of measles treatment, vaccine procurement, and vaccine delivery [37]. The results combined with procurement costs (estimated MR-MAP price multiplied by total MR-MAP doses for LIC and LMICs) are presented in terms of cost per measles disability-adjusted life year (DALY) averted for LMICs and compared to a cost-effectiveness threshold based on health opportunity costs. We explored two coverage scenarios: one in which routine coverage stagnates at 2019 levels and another in which it gradually increases to 95% two-dose coverage to meet global coverage targets.

### 2.3. Describing the Pathway from Development to Delivery

This section evaluated the potential challenges related to MR-MAP development and delivery. The iFVVA did not conduct any additional analyses related to MR-MAP characteristics, clinical development, or the required investment for development, as this is already being analyzed and actively monitored by the VIPS partnership [14,23].

#### 2.3.1. Identifying Key Product Characteristics for Decision-Makers

We interviewed regional immunization technical advisory group (RITAG) and national immunization technical advisory group (NITAG) chairs and members to better understand which product attributes would impact whether they would recommend the uptake of MR-MAPs. The interviews were structured around the TPP, and the respondents were asked to identify the five most important characteristics where evidence was required to determine if the MAP could be included in national immunization programs. The interview questions can be found in Table S5.

#### 2.3.2. Assessing the Technical Challenges to MAP Development

We conducted a rapid assessment of literature to collate data and lessons learned about the technical feasibility and potential challenges of developing microarray patches for the delivery of pharmaceutical drug treatments; see Table S2 for PubMed search. The results of the rapid assessment of literature were supplemented by interviews with MAP developers, vaccine manufacturers, and regulatory experts. Table S6 contains the interview questions.

#### 2.3.3. Conducting a Discounted Cash Flow Analysis

We conducted a discounted cash flow (DCF) analysis to calculate the indicative net present value (NPV) (a financial metric used to evaluate the profitability of an investment by calculating the present value of all future cash flows associated with that investment, both inflows and outflows, discounted back to the present using a specified rate) for a company investing in the development and manufacturing of MR-MAPs [38]. To calculate the NPV of the development project, key cash inflows and outflows were estimated, including revenues (calculated as demand multiplied by price), COGS, costs of clinical development, investment in a new manufacturing facility and its depreciation, sales, general, and administrative expenses, corporate taxes, and hurdle rate (the lowest rate of return a project or investment must achieve before a manager or investor deems it acceptable). The NPV is calculated per the following formula:

S_t=0_^n^ Net Cash Flow^t^/(1 + Hurdle Rate)^t^

where t = number of time periods (in this model, years).

We estimated the NPV from 2022–2040 assuming that the initial investments in clinical development and manufacturing scale-up would take place during 2022–2029. We assumed product launch in 2030, estimated revenue generation between 2030–2040, and assumed no foreign exchange fluctuations or inflation during the 11-year analytic period. To assess the robustness of our analysis and to account for the uncertainty in our model inputs, we conducted a univariate sensitivity. Table 3 provides an overview of the assumptions applied for the base scenario.

## 3. Results

### 3.1. The Global Need for Improved MR Vaccines and Ability of MR-MAPs to Overcome MR Problem Statements

**Figure 3:**
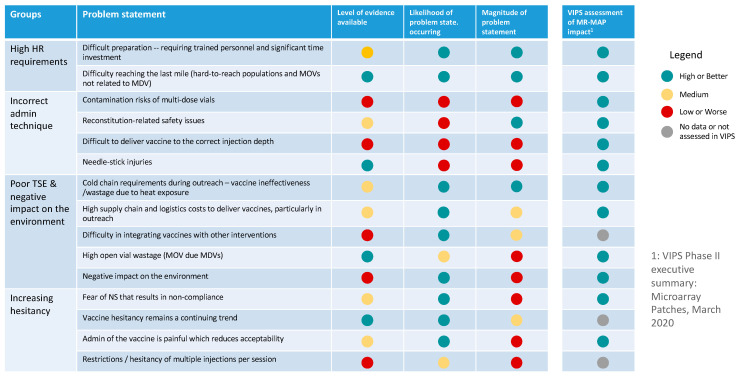
Summary of MR programmatic problem statements by level of evidence, likelihood of problem occurring, and magnitude of the problem statement.

Based on the rapid assessment of literature, 597 published articles and documents were identified, and of those, 125 were reviewed, resulting in the identification of 15 problem statements faced by immunization programs in delivering MR, which may impact the ability to effectively administer the lyophilized vaccine using an N&S (Figure 3). These programmatic problem statements were grouped into four categories: (i) high human resource requirements, (ii) ineffective administration procedures, (iii) poor total system performance and negative impacts on the environment, and (4) an increase in vaccine hesitancy. The problem statements with the greatest impact on immunization programs with the most evidence were related to high labor costs and challenges associated with equitably delivering N&S vaccines. See Table S7 for a summary of the evidence collated through the literature review on MR problem statements.

### 3.2. Estimating the Costs and Benefits of MR-MAP

#### 3.2.1. MR-MAP Use Cases and Demand Forecast

The prior work to develop the MR-MAP use cases identified six use cases that were utilized for the iFVVA [18].

Scenarios 3–6 explored potential MR-MAP demand considering variations on future coverage estimates and country introductions of MR-MAPs (scenarios 1 and 2 estimated potential demand if MR-MAPs were not made available and serve as the counterfactual). The MR-MAP demand forecast does not consider any supply, financial, or programmatic constraints and estimates the programmatic dose requirements (the average estimated number of doses a country would need to procure to meet its immunization program needs, whether these are routine or campaign; the requirement includes wastage, depending on the presentation, and buffer) (PDR) for the two scenarios of MR-MAPs, with the relative and stagnant coverage increase to be ~30 million doses increasing to ~230 million doses by 2040.

**Figure 4:**
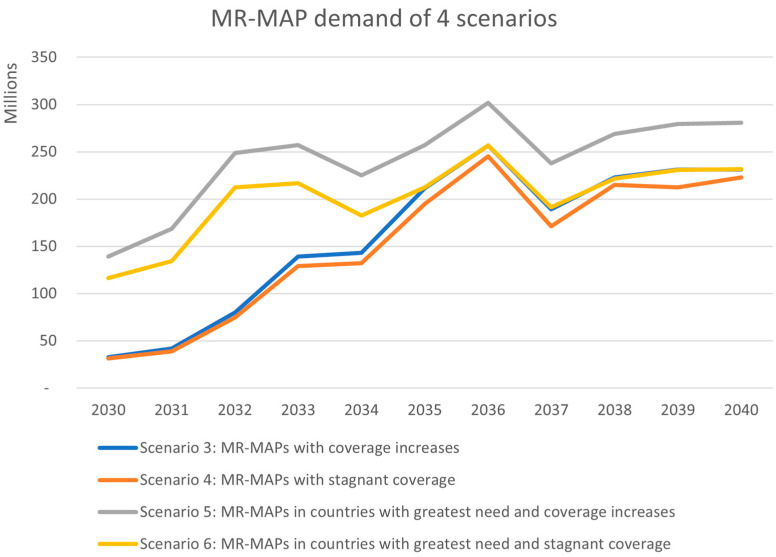
Results of MR-MAP demand for scenarios 3 to 6 (scenarios 1 and 2 estimated potential demand if MR-MAPs were not made available and serve as the counterfactual).

In comparison, scenarios 5 and 6 begin at a higher level of PDR in 2030 with ~140 and ~115 million doses, respectively. Scenarios 5 and 6 prioritize the earlier use of MR-MAPs in countries with the greatest public health need; it is unknown if these scenarios would materialize given the uncertainties in potential financial support and country introduction timing. The increase in PDR is driven by the earlier adoption of countries with the greatest burden and need. These countries are generally populous, and this ultimately increases MR-MAP PDR earlier in the forecasting period. Scenario 6 converges to a similar level of demand at ~230 million doses as scenarios 3 and 4 by 2040, driven by the lower coverage assumptions. However, scenario 5 remains higher at 280 million doses in 2040, due to the high coverage assumptions. Figure 4 provides a comparison of the demand from scenarios 3 to 6.

#### 3.2.2. Benchmarking an MR-MAP Price

Based on our review of the data sources, monovalent pediatric hepatitis B vaccine and pediatric pneumococcal conjugate vaccine (PCV) were selected as the most appropriate currently available benchmarks of price differential between MDV and SDV and SDV and PFS, respectively. The average price differential between MDV and SDV prices was 122%, and between SDV and PFS, it was 110%.

Using the 2021 MR MDV price procured by UNICEF for use in Gavi-supported countries ($0.58–$0.72 per dose), the average 2021 hepatitis B price differential of 122% was applied to obtain an estimated MR SDV price of $1.29–$1.60 per dose. To estimate a MR PFS price, the average 2021 PCV price differential of 110% was then applied to obtain an estimated price of MR PFS of $2.70–$3.36 per dose [30]. The average price differential between self-procuring LMICs and Gavi-supported countries of 15% was subsequently applied to obtain an MR PFS price of $2.35–$2.92 per dose for Gavi-supported countries. See Table 4 for an overview of the MR-MAP price benchmarks.

#### 3.2.3. Estimating the Commodity and Delivery Costs

The results of the commodity and delivery cost analysis indicate that MR-MAPs can be a cost-saving option for routine immunization (RI) if the optimal product characteristics (or MR-MAP Profile 1 in Table 2) are available. However, if this optimal MAP profile is not achieved, for the other profiles evaluated, MAPs could have higher commodity and delivery costs than the MAP N&S.

**Figure 5:**
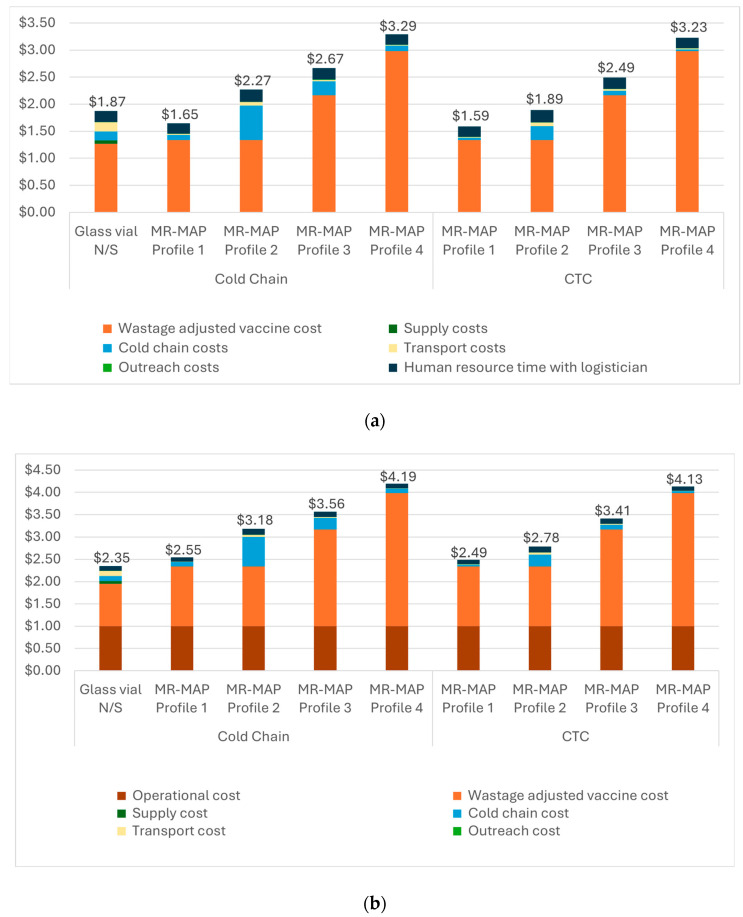
(**a**) Commodity and delivery costs for MR-MAPs by controlled temperature chain status for RI. (**b**) Commodity and delivery costs for MR-MAPs by controlled temperature chain status for SIA. For both RI (**a**) and SIA (**b**), the commodity costs accounted for the largest portion of the cost per dose administered. For RI settings, the next largest cost categories were cold chain costs and human resource time. For SIA settings, the next largest cost categories were the operational cost related to implementing SIAs and cold chain costs.

Figure 5a shows that MAP profile 1 could have a lower weighted average cost per dose administered than the current MR N&S ($1.59–$1.65 compared to $1.87) for routine immunization. While the vaccine price in MAP profile 1 is slightly more expensive than that of the N&S presentation, the reduction in vaccine wastage with the single-dose MAP presentation and the elimination of the syringes result in a lower commodity cost for MAPs. Commodity costs were the largest share of the total estimated cost per dose administered, accounting for 52% to 92% for the MAP compared to 67% for the N&S. In scenarios where the MR-MAP has higher vaccine prices, any delivery cost savings related to the smaller packaged volume or faster administration time were outweighed by the increase in the vaccine price.

When comparing the potential cost savings of CTC properties, our analysis indicates that CTC use could reduce the cost per dose administered by $0.06 (for MAP profiles 1 and 4) to $0.38 (for MAP profile 2), showing that the cost savings with CTC are greater when MAPs have a large packaged volume, as with MAP profile 2. Cost savings come from reduced cold chain costs when the CTC approach is utilized.

For SIAs, across all MAP profiles, MAPs are estimated to have a higher cost per dose administered than the N&S presentation (Figure 5b). Similar to routine immunization, the commodity costs accounted for the largest portion of the cost per dose administered in SIAs, ranging from 52–72% compared to 40% for the N&S presentation. Given the assumed lower wastage rate for the N&S in SIAs, the higher MAP price outweighed any potential savings from wastage reduction associated with the MAP’s single-dose presentation.

The next largest cost category was the operational cost related to implementing SIAs, which accounted for ~24–40% of the weighted average cost per dose administered for the MR-MAP product profiles compared to 43% for the N&S presentation. The costs for cold chain, transport, outreach, and HR accounted for ~5–26% of the total costs compared to 14% for the N&S presentation.

#### 3.2.4. Estimating the Potential Health Impact and Cost Effectiveness

With broad use for routine immunization and SIAs in LMICs, MR-MAPs were estimated to avert between ~6.9 and 7.5 million measles cases, 52,300 to 57,600 deaths, and 3.4 to 3.7 million DALYs over 2030–2040 under the scenario with estimated coverage increases. MR-MAPs are estimated to avert between 31.3 and 38.6 million cases, 390,000 to 402,000 deaths, and 25 to 26.3 million DALYs under the scenario with stagnant coverage growth in the future [37]. The stagnant coverage scenario assumes that 95% two-dose coverage cannot be attained using N&S presentations alone, and hence MAPs can have greater impact.

From a cost-effectiveness perspective, the cost per DALY averted for the low MR-MAP price estimate is between –$134 (cost saving) and $626 depending on country income status, while for the high MR-MAP price estimate, it is between $10.6 and $1846 [37]. The main cost driver was costs associated with measles treatment, which, if MR-MAPs were introduced in relatively higher income countries, would lead to cost savings given the reduced cost of treatment and potentially be cost-effective in 16–81% of LMICs [37]. The future performance of measles immunization programs and MAP procurement price played a key role in determining the cost-effectiveness, while the country introduction order and the discounting method had a limited effect [37]. Although cost-effectiveness will be context specific and depend on the final MR-MAP product attributes, our analysis indicated that MR-MAPs have the potential to be cost-effective across a diverse range of countries.

Lastly, the estimated cost per DALY averted is $85–$2310, which is comparable to the cost-effectiveness of vaccines in Gavi’s current portfolio, such as human papillomavirus (HPV) ($91–$928), rotavirus ($202–$428), and respiratory syncytial virus infection (RSV) maternal vaccines ($70–$270) [39,40,41].

More detailed results of the health impact modeling and cost effectiveness can be found in the published article [37].

### 3.3. Describing the Pathway from Development to Delivery

#### 3.3.1. Key Product Characteristics for Decision-Makers

**Figure 6:**
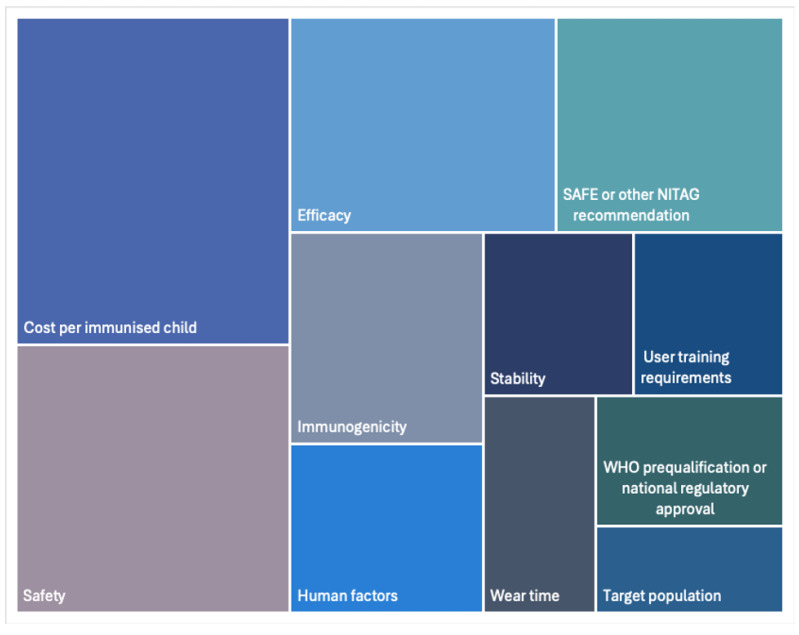
Overview of key product characteristics identified by decision-makers.

11 RITAG and NITAG chairs or members were interviewed to identify the main evidence needs for decision-making. The key evidence needs identified included cost per immunized child (*n* = 11), safety (*n* = 9), efficacy (*n* = 7), Strategic Advisory Group of Experts on Immunization (SAGE) or other NITAG recommendations (*n* = 6), immunogenicity (*n* = 5), and human factors (*n* = 4) as the main evidence needs for their decision-making. Figure 6 provides an analysis of the frequency with which specific evidence needs were stated by the interviewees.

#### 3.3.2. Technical Challenges to the Development MAPs

The search resulted in 35 published articles focused on the development of MAPs to administer pharmaceutical drugs for treatment of disease. Of the 35, 20 articles were reviewed in full, highlighting the outstanding questions that need to be addressed to enable the successful development and regulatory reviews of MR-MAP. These results were also validated in discussions with 26 individuals who were MAP developers, vaccine manufacturers, or experts in clinical development. The following challenges were identified:

- Generating immunogenicity data that shows non-inferiority and safety and reactogenicity data that shows comparable or reduced number of adverse events to the current N&S vaccine.
- Determining the correct M and R antigen dosage and developing analytical approaches to verify that an adequate quantity of antigen has been delivered to the patient.
- Improving the understanding of the optimization of bulk MR vaccine production.
- Determining the requirements for an aseptic (aseptic manufacturing is the process in which vaccine drug product and container are produced and combined in a completely sterile, self-contained environment) environment and/or process for manufacturing.
- Designing manufacturing processes using reproduceable methods that adhere to good manufacturing practices (GMP) to produce late-stage MR-MAP clinical trial materials and batch releases.
- Determining whether MR-MAP production will require a semi- or fully automated manufacturing line.
- Determining the thermostability and photostability of the MR vaccine in the MAP and appropriately reflecting it in the dossier, leaflet, etc. Note that the leaflet can also contain information regarding product ingredients.
- Developing regulatory guidance related to the design of MR-MAP clinical trials and at-scale manufacturing processes, including new quality assurance or quality control approaches.
- Conducting post-licensure evaluations (i.e., phase 4 studies) in different settings with variable temperature and humidity to further evaluate MR-MAP effectiveness and consistency.

#### 3.3.3. Conducting a Discounted Cashflow Analysis

**Figure 7:**
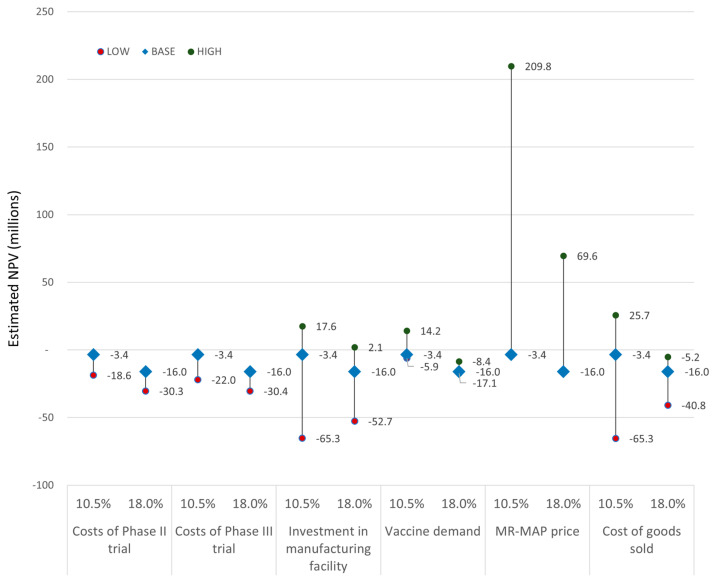
Estimated NPV by scenario assuming a hurdle rate of 10.5 and 18%. See Table 3 for details on overview of the assumptions applied for the Base case as well as the Low and High DCF scenarios modelled.

The estimated NPV for the different scenarios is shown in Figure 7 for two different hurdle rates. In the base case scenario, we estimated an NPV of −$3.4 million and −$16.0 million, assuming a hurdle rate of 10.5% or 18%, respectively.

For the 10.5% hurdle rate, which reflects a higher risk tolerance, a positive NPV was achieved with higher MR-MAP price (+$209.8 million), lower COGS (+$25.7 million), lower investments in the manufacturing facility (+$17.6 million), and higher vaccine demand (+$14.2 million). A higher cost of phase II (−$18.7 million) and III (−$22.0 million), higher investments in manufacturing facility (−$65.3 million), lower vaccine demand (−$5.9 million), and higher COGS (−$65.3 million) resulted in negative NPVs.

For the 18% hurdle rate, which reflects a lower risk tolerance, a positive NPV was achieved with the higher MR-MAP price (+$69.6 million) and lower investments in the manufacturing facility (+$2.1 million). A higher cost of phase II (−$30.3 million) and phase III (−$30.4 million), higher investments in manufacturing facility (−$52.7 million), and higher COGS (−$40.8 million) resulted in negative NPVs. The vaccine demand scenarios did not result in a positive NPV (−$17.1 to −$8.4 million).

## 4. Discussion

### 4.1. The Global Need for Improved MR Vaccines and the Ability of MR-MAPs to Address the MR Problem Statements

The iFVVA highlighted that the problem statements with the highest likelihood of occurrence and the greatest impact on MR immunization programs are related to high labor costs, challenges associated with equitably delivering N&S vaccines, and challenges with cold chain requirements during outreach activities. For these problem statements, it is anticipated that MR-MAPs’ single-dose presentation and ease of preparation and administration could expand the vaccinator workforce to community-trained health workers or volunteers, ultimately reducing labor costs and providing opportunities to vaccinate previously unreached children. Further MR-MAPs are anticipated to have more thermostable characteristics compared to the current N&S vaccine (e.g., vaccine vial monitor of 30 days and CTC properties), which can be made to address the cold chain challenges during outreach activities, which may also positively contribute to more equitable MR vaccination coverage.

As it is anticipated that MAPs can address some of the problem statements, it would be important that evidence relating to how MR-MAPs can impact these problem statements is generated and translatable to different countries and contexts.

Recent phase I and I/II studies found that MR-MAPs were safe and well-tolerated with promising immunogenicity results, and the most common side effects with MAPs are local reactogenicity at the application site [42,43,44]. Although MR-MAPs could offer several potential benefits, their successful implementation will require appropriate training, development of implementation strategies, and adequate infrastructure adaptation; as well as community sensitization to educate the community about MAPs and to promote acceptability.

Thus, MR-MAPs will likely require implementation research studies as well as large-scale use to better understand the potential programmatic impact of a MAP presentation. Global partners under leadership of WHO and a technical advisory group are addressing this by defining an implementation research agenda, so that such data become available with minimal delays [45].

### 4.2. Estimating the Costs and Benefits of MR-MAPs

While the price benchmarking analysis estimates the Gavi-supported price in the range of $1.29–2.92, the actual price will depend on MR-MAP COGS, the scale of manufacturing, the options for a multi-purpose facility, and individual country use and the balance of supply and demand. Moreover, it is unlikely that the MAP price will be lower than the theoretical costs of the MR SDV presentation ($1.29 in Gavi-supported countries) given the need for developers to recover upfront costs for clinical development and manufacturing scale-up. Finally, due to the low cost of existing MR N&S presentations, potential buyers of MR-MAPs may be highly sensitive to any increases in price. Since the development of this iFVVA, an initial estimate of targeted MR-MAP demand has been developed to take into account how MR-MAPs could be deployed to limit the financial burden linked to the initial expected price premium associated with MR-MAPs compared to the MR MDV presentation; however, it is essential that this is further explored to consider country perspectives.

Unsurprisingly, for the commodity and delivery costs analysis, the MR-MAP price was the largest driver, and so potential variations in the cold chain volume, HR time to administer the MR-MAP, and CTC qualification had a smaller impact on the cost estimates. As MR-MAP development advances towards market availability, balancing the appropriate price will ensure country and manufacturer sustainability. Furthermore, optimizing the key product characteristics of smaller cold chain volume and CTC will have an impact on delivery costs and ultimately the value proposition. The analysis also found that given the high wastage rate for multidose MR N&S, the single dose presentation of MR-MAPs reduces wastage, and the cost savings are greater for RI than SIAs, where wastage rates with the current multi-dose vial presentations are higher. Finally, the analysis showed marginal cost savings due to shifting the vaccinator workforce to HCWs, given relatively low costs of labor in LMICs.

The iFVVA was the first analysis to estimate the public health impact of MR-MAPs on measles disease burden well as its cost effectiveness, highlighting up to a 35% reduction in measles cases, deaths, and DALYs while showing MR-MAPs to be a cost-effective intervention across most of the LMICs under stagnant coverage [37]. On the other hand, in countries with a higher income, introducing MR-MAPs had the largest cost savings due to prevention of measles treatment costs. These results indicate that MR-MAPs could have broader impact and cost effectiveness beyond the low-income countries, highlighting the potential for a dual market. The cost-effectiveness of MR-MAPs is expected to further increase if more targeted use of MR-MAPs is implemented and/or increased equity in coverage is valued higher and/or if there is earlier elimination of the measles disease and the related economic burden. It is anticipated that this will further increase the value for money of MR-MAPs, making it one of the most cost-effective vaccine interventions.

Furthermore, the iFVVA demonstrated that MR-MAP cost per DALY saved for LMICs ($85–$2310) is comparable to the HPV, rotavirus, and RSV maternal vaccines, indicating that MR-MAPs could have good value for money and are comparable to historical donor-led investments in vaccines [39,40,41].

While the results of these analyses are promising and highlight the potential benefits of implementing MR-MAPs, the demand forecast for MR-MAPs serves as a key input that assumes broad uptake of MR-MAPs and the ability to reach un- and under-vaccinated children. On top of developing more targeted use scenarios, assumptions on uptake by countries and ability to reach un- and under-vaccinated children need to be further developed and validated. Furthermore, as MR-MAPs advance into late-stage clinical development, it would be important to refine these analyses with more accurate price estimates.

The analyses did not consider the potential impact of coexisting interventions apart from vaccination as well as whether the increase in coverage could achieve MR elimination faster and result in changes in vaccination strategies, such as fewer SIAs or fewer outbreak responses. Linked to this, it would also be important to capture any potential cost savings related to these potential changes in vaccination strategies. These limitations of the public health impact and cost-effectiveness analysis may result in an under-estimate of the impact of MR-MAPs and should be explored as part of future investment cases.

The analysis did not include the environmental impact of MAPs, and this should be an additional focus for future analysis. The environmental impact of manufacturing MAPs will depend on factors such as cold chain volume, weight, and material selection. MAPs will have a significant impact on reducing open-vial wastage as well as reducing waste at the end of product life (e.g., no glass vials, needles, or syringes) and reducing CO_2_ emissions.

### 4.3. Describing the Pathway from Development to Delivery

The iFVVA captured feedback from RITAG and NITAG members, highlighting the need to not only understand key product characteristics such as safety, efficacy, and immunogenicity but to also have evidence on cost per immunized child and recommendations from SAGE or RITAG. However, this analysis only consulted 11 individuals, and it would be important to continue these conversations with decision-makers as MR-MAPs advance in their development. The rapid assessment of literature provided information on the potential challenges to successfully developing and manufacturing MR-MAPs. These development challenges are being actively monitored by the VIPS partners and will require coordination between MR-MAP developers and regulators to support timely marketing authorization and implementation in LMICs.

Despite the high public health potential of MR-MAPs, our analysis indicates that the investment required to develop them will not be attractive unless concerted actions are taken by global health stakeholders. The factors that have the highest impact on the financial sustainability are the differential between price and COGS, the manufacturing set-up, and the financing of late-stage clinical development. These data are critical to quantify the financial gap that must be covered to allow for the development and production of MR-MAPs. It is likely that this un-recouped investment will have to be covered by third parties via different instruments impacting the most relevant variables, such as a top-up on price, demand guarantees, and/or an upfront contribution to the clinical development and manufacturing investments.

### 4.4. Benefits of the iFVVA

The FVVA methodology provided flexible guidance on the key analyses and areas that are required to better understand the full value of MR-MAPs [21]. The iFVVA aims to take an early and holistic approach to understanding the value of MR-MAPs through conducting analyses important to the public health stakeholders, decision- and policy-makers, and MAP developer/vaccine manufacturer perspectives. Thus, the iFVVA served as an excellent tool to align stakeholders on key assumptions and explore the areas of uncertainty using scenarios and sensitivity analyses. The iFVVA also opened discussions to others interested in MR-MAPs who may not typically be consulted at this stage, such as vaccine manufacturers, MAP developers, donors/funders, regulatory experts, procurement agencies, and regional and national level decision-makers.

MR-MAPs were not yet in Phase I trials when the iFVVA development began, and thus this assessment aimed to identify the outstanding questions considering the public health and commercial perspectives. Furthermore, while the iFVVA provided additional analyses to support the MR-MAP development discussions, it was completed early in the development of MR-MAPs, with some assumptions being driven by expert opinion. The analyses presented in the iFVVA will need to be updated as new data on country use, demand, price, and product characteristics become more concrete. Such analyses may include more targeted approaches on use cases to identify the highest added value of MR-MAPs and thus the best return on investment in the MR-MAPs, considering the potential price, the public health return, and countries’ and donors’ willingness to pay.

Although there is significant hope around MR-MAPs, their implementation and delivery will depend heavily on generating additional evidence and research on their benefits, as well as if there are innovative strategies in which MR-MAPs could be used to obtain high public health impact. Implementation research and potential pilots/studies will need to be conducted to generate sufficient evidence to assist in the global, regional, and national decision-making processes.

The iFVVA generated novel analyses; however since its development, two MR-MAP products have completed Phase I/II and Phase I studies, indicating the need to regularly update analyses as more data become available. Furthermore, additional analyses not explored in the iFVVA may also prove important in analyzing its full value. These analyses include the potential public health impact on the rubella disease burden and the potential changes in vaccination strategy if MR-MAPs help in achieving and sustaining 95% coverage goals (e.g., fewer SIAs, less outbreak response, and less measles and rubella burden). Moreover, while the epidemiological and economic modeling provides estimates of the health impact and cost-effectiveness of MR-MAPs across large income groups, detailed country-specific modeling is needed to inform country-specific strategies for MR-MAP introduction. Finally, the environmental impact of MAPs should be an additional focus for future analysis.

## 5. Conclusions

The iFVVA shows the high potential public health impact and the cost effectiveness of MR-MAPs, particularly related to increasing equitable vaccine coverage among un- and under-immunized populations as well as contributing to achieving and sustaining measles elimination goals. The public health impact will be larger the earlier MR-MAPs are introduced and used. Regardless of its high public health potential, MR-MAPs have not yet completed critical clinical, regulatory, and manufacturing milestones and require more evidence generation on outstanding implementation questions. All these factors could contribute to the delay of MR-MAP use in LMICs. Due to the variety of factors impacting the potential timelines of MR-MAPs, a coordinated effort across a range of stakeholders including MAP developers, vaccine manufacturers, donors, and financiers, as well as policy- and decision-makers at the global, regional, and national levels, will be needed to ensure timely development and use in LMICs. The iFVVA provided an initial opportunity to bring together key stakeholders and discuss the areas of uncertainty and identified potential actions that could drive the acceleration of MR development and implementation. The iFVVA identified the following key actions that will require coordinated efforts from all but should be led by the following key stakeholders (note that there is ongoing work for the actions marked with ⬤ *i*):

### 5.1. MAP Developers and Vaccine Manufacturers

- Accelerate partnering and collaboration of the development of MR-MAPs, as well as other vaccine-MAPs.
- Build in key programmatic needs in the MAPs development programs, such as minimized COGs, minimized cold chain volume, increased thermostability (CTC and VVM30), and minimized wear time.
- Factor in sustainability directions, including minimizing COGs and exploring the potential for multiple MAP manufacturing facilities/production lines, local manufacturing, and dual market (LMIC, UMIC, and HIC) opportunities.

### 5.2. Donors and Financiers

- Advance the technical maturity of the MAPs platforms, including the optimizing MAP designs/characteristics and the availability of manufacturing facilities.
- Prove the technical feasibility of MAPs as a vaccine delivery platform to increase commercial sustainability.
- Share upfront cost of MAP developers and vaccine manufacturers, especially development costs, manufacturing investments, and late-stage clinical trials.
- Wxplore cost sharing of MR-MAP development with more commercially attractive vaccine-MAP products (e.g., seasonal influenza, measles-mumps-rubella, measles-mumps-rubella-varicella).
- Continue to refine, qualify, and quantify the commercial value of MR- and other vaccine-MAPs.

### 5.3. Policy- and Decision-Makers at Global, Regional, and National Levels

- Develop and contribute to WHO Evidence Considerations for Vaccine Policy (ECVP) in identifying additional data requirements for SAGE review and implementation of research questions.⬤
- Identify and prioritize implementation research questions, which will further enrich the existing knowledge and prepare for programmatic introduction. ⬤
- Collaborate with countries, including Gavi-supported and self-procuring countries, to design and conduct post-licensure implementation studies, including those to evaluate MR-MAP’s ability to reach un- and under-vaccinated children, as well as better quantifying potential demand.
- Drive development of global, regional, and national guidance and policies to include MR-MAP into immunization programs and explore potential administration by lesser trained health workers or self-administration, including for HICs to explore the use of MR-MAPs in sustaining MR elimination.
- Support the development of financing policies to support MR-MAP implementation, including Gavi eligibility/co-financing.
- Define use and training needs for MR-MAPs that are potentially used co-currently with MR N&S.
- Refine iFVVA analyses to inform future investment decisions as MR-MAPs continue advancing in their development, including the following:
  (a) The use cases to consider programmatic “fit-for-purpose” to inform future vaccination strategies at the subnational level;
  (b) The demand estimates, including estimating targeted introduction (e.g., use in a subset of countries/regions and/or use cases or delivery strategies) of MR-MAPs ⬤;
  (c) The public health impact and cost effectiveness, for both measles estimates and rubella estimates, informed by implementation research ⬤;
  (d) The economic analyses expanding the beyond health opportunity costs to include the value of economic value of productivity gains, socioeconomic equity around improved coverage, environmental sustainability (e.g., CO_2_ footprint, waste), the impacts of earlier measles and rubella elimination (e.g., the reduced number of measles outbreaks and health system disruptions, reduced need for follow-up campaigns);
  (e) The DCF analysis to revise the commercial viability and identify potential market-shaping interventions.
- Design a risk-sharing mechanism for MAP development, scale-up, and implementation in LMICs to balance the financial pressure on developers and manufacturers, such as demand guarantees or a price top-up.
- Support potential partnerships between MAP developers and vaccine manufacturers.

Actions have been discussed in the External Advisory Group of the iFVVA, and, consequently, the represented stakeholders are undertaking the *italicized* activities. This includes coordination via VIPS and the Technical Advisory Group for MR-MAPs. While we have identified several key actions that need to be led by various stakeholders, the main underpinning of the successful development and implementation of MR-MAPs relies on continuous and open discussions amongst all key stakeholders.

## Figures and Tables

**Table 1:** Overview of scenarios utilized for the iFVVA.

#	Name	Presentation(s) Modeled		Adoption Timing of MR-MAPs		Coverage Assumption Used	
		N/S	MAPs	No Weighting	Countries in Greatest Need Introduce Earlier	High	Low
1	No MR-MAPs with coverage increases	x				x	
2	No MR-MAPs with stagnant coverage	x					x
3	MR-MAPs available with coverage increases	x	x	x		x	
4	MR-MAPs available with stagnant coverage	x	x	x			x
5	MR-MAPs are implemented in countries with greatest need with coverage increases	x	x		x	x	
6	MR-MAPs are implemented in countries with greatest need with stagnant coverage	x	x		x		x

**Table 2:** Key assumptions for estimating commodity and delivery costs ^1^.

MR Vaccine	N&S			MAP			
	1-Dose	5-Dose	10-Dose	Profile 1	Profile 2	Profile 3	Profile 4
**Estimated price per dose** **^2^**	$2.48	$0.90	$0.72	$1.29	$1.29	$2.11	$2.92
**Doses per vial**	1	5	10	1	1	1	1
**Volume of the vaccine per dose (cm^3^), primary and secondary packaging**	21.09	4.218	2.109	3	20	8	3
**Human resource time use (in seconds) for vaccine administration**	48	35	21	20	200	120	20
**RI vaccine wastage rate**	5%	15%	40%	1%	1%	1%	1%
**SIA vaccine wastage rate**	1%	10%	10%	1%	1%	1%	1%
**Volume of diluent per dose (cm^3^)**	12.53	5.48	3.14	0	0	0	0

^1^ Each MAP profile was modeled separately with and without CTC use, where without CTC use assumes that MAP is in the cold chain for the entire supply chain until vaccine administration. In the commodity and delivery costs results, the average of the 5- and 10-dose vial presentations are presented together as the comparator presentation. ^2^ Prices per dose for N&S presentation were derived from the price benchmarking analysis.

**Table 3:** Overview of DCF scenarios.

Variable	Base Case	Scenarios Modelled
**Vaccine demand** [17]	MR-MAPs are adopted beginning in 2030 with the high coverage assumption No HIC demand is included Assumes significant uptake of MR-MAPs to 100 million doses	HIC demand is included Assumes lower uptake of MR-MAPs to 50 million doses
**Market prices for MR-MAPs**	Gavi: $1.29 Self-procuring LMIC: $1.48 UMIC: $2.63 HIC: $9.11	Higher estimated MR-MAP prices were used: Gavi: $2.92 Self-procuring LMIC: $3.36 UMIC: $5.20 HIC: $20.64
**COGS**	30% mark-up on the Gavi price or $0.90	$0.90 and declines to $0.70 starting in Year 6 Upper bound of $1.40
**Clinical development cost**	Phase I: Fully financed by the donor Phase II: $24 million and financed by a donor Phase III: $18 million from 2025–2027	Phase I: Fully financed by the donor Phase II: $16.8 million Phase III: $45.5 million
**Manufacturing facility investment**	A required investment of $60 million for the MAPs filling line setup: $30 million small-scale plant and $30 million for scale up to 100 million dose capacity (fill and finish only)	A required investment of $30 million for scale up to 100 million dose capacity (fill and finish only) A required investment of $37.5 million for a small-scale plant and $173 million for expansion to 100 million dose capacity (including drug substance)
**Hurdle rate (Ibid)**	10.5%	18%

**Table 4:** MR-MAP price benchmarks for Gavi-supported countries.

	MR MDV (A)	Hep B Price Differential (B)	MR SDV C = A + (A × B)	PCV Price Differential D	MR PFS E = C + (C × D)
**Lower bound**	$0.58	122%	$1.29	110%	$2.70
**Upper bound**	$0.72		$1.60		$3.36

## Data Availability

The raw data supporting the conclusions of this article will be made available by the authors on request.

## Supplementary Materials

The following supporting information can be downloaded at https://www.mdpi.com/article/10.3390/vaccines12091075/s1: Figure S1: Current MR-MAP development timeline; Table S1: Expert Advisory Group members; Table S2: PubMed search terms; Table S3: Formula to estimate MR PFS price in Gavi-supported countries; title; Table S4: Additional assumptions to estimate the commodity and delivery costs; Table S5: Interview questions for policy- and decision-makers; Table S6: Interview questions for MAP developers and vaccine manufacturers; Table S7: Summary of evidence on each measles and rubella problem statement evaluated in problem statements assessment [7,10,46,47,48,49,50,51,52,53,54,55,56,57,58,59,60,61,62,63,64,65,66,67,68,69,70,71,72,73,74,75,76,77,78,79,80,81,82,83,84,85,86,87,88,89,90,91,92,93,94,95,96,97,98,99,100,101].
