# Supplementary material for: An Application of an Initial Full Value of Vaccine Assessment Methodology to Measles-Rubella MAPs for Use in Low- and Middle-Income Countries
Source: Vaccines (Basel). 2024 Sep 19;12(9):1075. doi: 10.3390/vaccines12091075 (PMC11435702; doi:10.3390/vaccines12091075)
Supplement: Supplementary file 1 [file vaccines-12-01075-s001.zip › vaccines-3183623-supplementary.pdf]

SUPPLEMENTARY MATERIALS

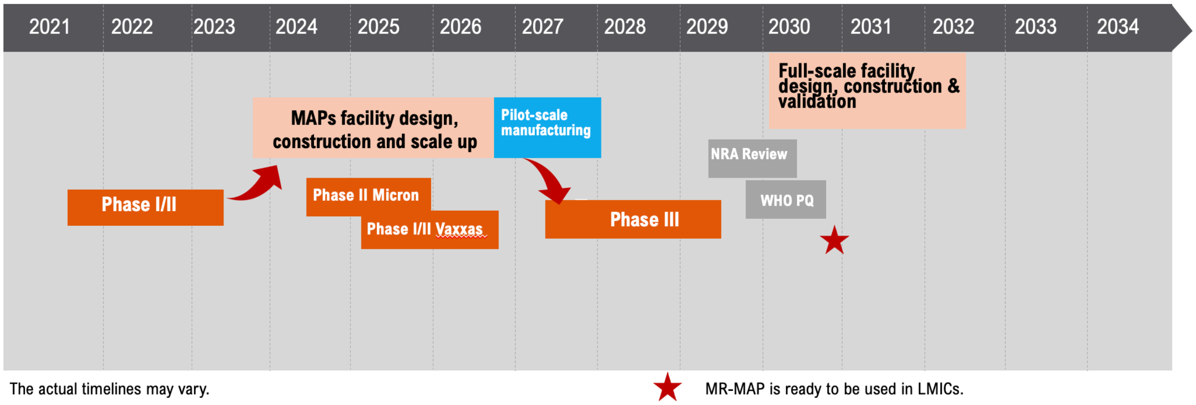

Figure S1. Current MR-MAP development timeline.

Table S1. Expert Advisory Group members.

UNICEF convened an Expert Advisory Group to provide their strategic feedback and direction on the development of the iFVVA. The Expert Advisory Group members bring different perspectives of global, regional, and national level policy- and decision-making as well as MR implementation, emergencies and humanitarian, vaccine clinical development, regulatory, manufacturing, pharmaceutical, financing, and procurement experiences and perspectives. The Expert Advisory Group members also included focal persons from the VIPS partnership. The members are listed in Table S1.

Name	Organization
Narendra Arora	INCLEN, India
Anindya Bose	WHO
Ma Chao	China CDC
Natasha Crowcroft	WHO
Anh Dang	MOH-Vietnam
David Durrheim	U of Newcastle
Birgitte Giersing	WHO
Philipp-Alexandre Gilbert	BMGF
Courtney Jarrahian	PATH
Lidia Kayembe	US CDC

Melissa Malhame	Adjuvance Tech
Marion Menozzi-Arnaud	Gavi
Chris Morgan	JHPIEGO
Daniel Rodriguez	PAHO
Glenn Rockman	Adjuvant Capital
Sally Nicholas	Wellcome
Monica Rull	MSF
Alka Sharma	BIRAC/DBT
Philipp Lambach	WHO IVR

Table S2: PubMed search terms

Rapid assessment of literature	Search terms	Date
MR problem statements	(i) (Measles OR rubella) AND challenge<br>(ii) (Measles OR rubella) AND (cost or impact); and<br>(iii) Measles or Rubella AND (Delivery issue)	Jan 2015 to Apr 2021
MAP development challenges	((((microarray patch) OR (microneedle patch)) AND (drugs)) AND (disease))	Jan 2019 to Apr 2021

Table S3: Formula to estimate MR PFS price in Gavi-supported countries

Input	Formula component
10-dose vial MR price per dose for Gavi-supported countries (2021)	a
Average price difference between MDV and SDV for Gavi-supported countries (%)	b
Theoretical MR SDV price per dose for Gavi-supported countries	c=a+(a x b)
Average price differential: SDV and PFS for self-procuring LMICs.	d
Theoretical MR PFS price per dose for self-procuring LMICs	e=c+(c x d)
Average price differential: Self-procuring LMICs vs. Gavi-supported countries	f
Theoretical MR PFS price per dose of for Gavi-supported countries	g=e/(1+f)

Table S4: Additional assumptions to estimate the commodity and delivery costs[1]

MR vaccine	N&S			MAP			
	1-dose	5-dose	10-dose	Profile 1	Profile 2	Profile 3	Profile 4
Volume of injection syringe (cm^3)	42.83	42.83	42.83	0	0	0	0
Cost of injection syringe	$0.04	$0.04	$0.04	$0	$0	$0	$0
Volume of reconstitution syringe (cm^3)	34.30	34.30	34.30	0	0	0	0
Cost of reconstitution syringe	$0.04	$0.04	$0.04	0	0	0	0
Wastage rate for syringes (for vials only) and or safety boxes	10%	10%	10%	10%	10%	10%	10%

Table S5: Interview questions for policy- and decision-makers

#	QUESTIONS
1.	Please provide an overview of the NITAG that you currently chair (e.g., size and membership, is there a representative from NRA, how often does it meet, high-level description of the process used for decision-making/how are agendas set, what is the relationship with RITAGs), what are the key data points and considerations that enabled the NITAG to make its most recent recommendation(s), how is the quality of evidence evaluated (e.g., GRADE or ACIP framework), are working groups utilized?).
**2.**	Has the NITAG made any recommendations on the uptake of new presentations and how was that recommendation made (e.g., 5-dose vial of MR versus 10-dose vial, PCV13 versus PCV10)? Are there any required data points or evidence (e.g., cost effectiveness analysis) prior to making a recommendation?
3.	With reference to the MR-MAP Target Product Profile (see table 1 below), from your perspective as NITAG chair, please identify the 5 most important or least important characteristics likely to impact a NITAG's recommendation for MR-MAPS.
4.	Please explain your rationale for choosing these specific characteristics and could you please prioritize these characteristics.
5.	Are there other characteristics, criteria, or data that you think are essential for the NITAG to make a recommendation on a MR-MAP product?
6.	What are the barriers to getting a NITAG recommendation for use, and are there any barriers for your country to use the new product following a NITAG recommendation?
7.	Please add any other questions or comments.

[1] Each MAP profile was modelled separately with and without CTC use, where without CTC use assumes that MAP is in the cold chain for the entire supply chain until vaccine administration. In the commodity and delivery costs results, the average of the 5- and 10-dose vial presentations are presented together as the comparator presentation.

Table S6: Interview questions for MAP developers and vaccine manufacturers

#	QUESTIONS
1.	What specific aspects do you see as especially important in the clinical development of a vaccine with a MAPs presentation (e.g., clinical endpoints, safety, trial design, etc.)?<br><br>(Probe: if there are specific considerations for MR-MAPs)
2.	What specific safety requirements would need to be met by a vaccine with a MAP presentation during clinical development?<br>(Probe: whether separate safety data would be required both for reformulated MR and the MAP device; if safety requirements would differ for unique MAP technologies – coated, dissolvable, etc.)
3.	What is your view regarding the duration of the full clinical development process for a vaccine with a MAP presentation compared to a standard needle and syringe presentation? Are there ways the MAP development timeline could be accelerated?<br><br>(Probe: which steps of will take more/less time for MAP compared to traditional vaccine development and if MR-MAPs has specific considerations which may impact timeline)
4.	Are there lessons from COVID-19 vaccine development that can be applied to accelerate MR-MAP development?
5.	Would probability of success of MR-MAP be higher than a similar “traditional” EPI vaccine? Would existing evidence of safety and efficacy of needle-syringe MR vaccines affect the probability of success?<br><br>Note: Probability of success of a vaccine candidate to advance from preclinical to clinical usually ranges between 40% and 50% but to be successful through phase 2 the probability goes down to only 10–13% (doi:10.1080/21645515.2019.1629254).<br><br>(Probe: if standard estimates of probability of success for vaccine development need to be modified for vaccine with a MAP presentation; if there are stakeholders who may be more willing to accept any increases in risk to develop MR-MAP and why?)
6.	What are the key challenges and requirements of manufacturing MR-MAPs compared to the traditional (multi dose) vial presentations (e.g., separate diluent)? Do you see any key trade-offs?<br>Are these challenges the same across all types of MAPs technologies (e.g., coated, dissolvable)? Are there clear potential solutions to these challenges?
	Do you foresee significant changes in the cost of producing MR-MAPs vs N&S presentation?<br>(Probe: which production steps are more impacted and the associated cost implications)
7.	What is the anticipated/expected and most feasible/cost effective manufacturing scale-up scenario for MR-MAPS? Potential scenarios include:<br>• Phase 3 clinical trial material and pilot introductions<br>• Commercial plant with low level capacity (e.g., 10m doses/year)<br>Commercial plant with high level capacity (e.g., 50m doses/year)
8.	Is it feasible to develop a MAP manufacturing platform to produce multiple vaccines (in/on MAPs) within the same manufacturing facility? Which vaccines would be most feasible to produce in the same MAP-“F&F”-facility?
9.	Are there historical partnership models on clinical development, both technical and financial, that could be considered good templates to support MR-MAPs development?

10.	What factors drove success of those partnerships? Are those transferrable to the development of MR-MAPs?
11.	When, within the timeframe of clinical development, should the establishment of a commercial partnership be completed?<br><br>(Probe: if there are potential timeline impacts depending on timing of clinical development partnership)
12.	Are you aware of commercial partnerships between MAP developers and vaccine manufacturers? Do you have an understanding of the nature of these partnerships (e.g., joint venture, tech transfer, CMO)?
13.	In your view, what types of vaccine manufacturers or pharmaceutical companies would be most interested in commercializing vaccines in/on MAPs? And for MR-MAPs specifically?
14.	Any other comments or points you would like to add.

Table S7: Summary of evidence on each measles and rubella problem statement evaluated in problem statements assessment

Category	Potential impact on MR goals and objectives
**High human resource requirements**	• Average time across 20 health workers to delivery one dose of a 10-dose lyophilized vial was 20.9 seconds and one dose lyophilized vial was 48.3 seconds [46]<br>• It is estimated that there are over 22m zero-dose children and over 18m under-vaccinated children.[3]) Historically the number of zero dose children has not decreased over time and has remained stagnated at ~20 million children since 2010.<br>• A systematic review of MOV since 1993 indicates that MOV prevalence was among 32% among children; however MOV considers timeliness and in the majority of these cases, the children are vaccinated but generally at a later age than the recommended immunization schedule.[47]
**Incorrect administration technique**	• Reconstitution errors occurred in more than 1 per 100 vaccinations. [7]<br>• Bacterial contamination was identified in 6 out of 3640 (0.2%) MDVs [48]<br>• The average number of injuries per health worker (0.2–4.7 sharps injuries per year). [49-53]<br>• The annual proportions of health workers exposed to bloodborne pathogens was 2.6% for Hepatitis C viral, 5.9% for Hepatitis B viral and 0.5% for HIV, corresponding to about 16,000 Hepatitis C viral infections and 66,000 Hepatitis B viral infections in health-care workers worldwide. [49-53]<br>• There is no evidence quantifying the impact of vaccine ineffectiveness at incorrect injection depth.
**Poor TSE and negative impact on the environment**	• Exposure to heat has relatively limited per the published literature and likely not to have significant impact on the overall ability to reach programmatic goals, but if MAPs have CTC characteristics it can reduce vaccine ineffectiveness due to heat exposure as well as provide potential cost savings related to outreach activities.<br>• Interviews with countries indicated that conducting outreach activities can be time consuming and costly endeavour.<br>• Limited information on how best to integrate vaccines with other health interventions and the potential cost savings [54-56]<br>• Reported open vial wastage is at or higher than indicative rates for both 5- and 10-dose vials at 15% and 40%, respectively. For 5-dose vials ranges the published literature indicates wastage rates to be from 16%-30% [15, 57-62]

	• If countries utilizing 10-dose vials were to adopt MR-MAPs they would experience ~39% decrease in the number of doses needed, while countries utilizing 5-dose vials could experience a decrease in 14%.[2]1<br>• MOVs can be caused by HWs not willing to open large dose vials due to high open vial wastage [47, 63-74]<br>• There is a negative impact of N&S waste on environment [75]
**Increasing vaccine hesitancy**	• Vaccine hesitancy has major implications on programmes and vaccination coverage [76-91], one study in LMICs and LICs has been published that evaluates the end-user acceptability of vaccine-MAPs in children, which showed a high level of acceptability of 92.7% from the 314 people who participated in the study. [10] Additional studies have been conducted which indicate a high level of acceptability by healthcare professionals and caregivers by but these studies occurred in HICs. [92-95] There is also ongoing work to examine the acceptability of MR-MAPs as part of the Phase I trials.<br>• Estimates on the fear of needles in the total population is ~5-10% impact. Literature is focused on HICs, and inconclusive if it leads to non-compliance with routine vaccination [96-101]<br>• Limited literature on potential perceived hesitancy to deliver multiple injections in one session or potential restrictions on delivering multiple vaccines in one session.

[2] Assumes WHO indicative wastage rates of 40% for 10-dose vial and 15% for 5-dose vial and 1% wastage rate for MR-MAPs.
